# CD20^+^CD22^+^ADAM28^+^ B Cells in Tertiary Lymphoid Structures Promote Immunotherapy Response

**DOI:** 10.3389/fimmu.2022.865596

**Published:** 2022-05-11

**Authors:** Zhenghao Wu, Junjie Zhou, Yunxiao Xiao, Jie Ming, Jing Zhou, Fang Dong, Xiaoqi Zhou, Zhuoshuo Xu, Xiangwang Zhao, Ping Lei, Tao Huang

**Affiliations:** ^1^ Department of Breast and Thyroid Surgery, Union Hospital, Tongji Medical College, Huazhong University of Science and Technology, Wuhan, China; ^2^ Department of Immunology, School of Basic Medicine, Tongji Medical College, Huazhong University of Science and Technology, Wuhan, China; ^3^ Department of Pathology, Union Hospital, Tongji Medical College, Huazhong University of Science and Technology, Wuhan, China

**Keywords:** B cells, tertiary lymphoid structure, immunotherapy, ADAM28, immune checkpoint inhibitors

## Abstract

**Background:**

As the indication for immunotherapy is rapidly expanding, it is crucial to accurately identify patients who are likely to respond. Infiltration of B cells into many tumor types correlates with a good response to immune checkpoint inhibitor (ICI) therapy. However, B cells’ roles in the anti-tumor response are far from clear.

**Methods:**

Based on single-cell transcriptomic data for ICI-treated patients, we identified a B-cell cluster [B_IR_ (ICI-Responsive B) cells] and described the phenotype, cell–cell communication, biological processes, gene signature, and prognosis value of B_IR_ cells through bioinformatic analysis, tissue immunofluorescence, and animal experiments. Surgery samples from 12 non-small cell lung carcinoma (NSCLC) patients with adjuvant checkpoint blockade were evaluated as external validation.

**Results:**

B_IR_ cells were identified as a subset of CD20^+^CD22^+^ADAM28^+^ B cells with a memory phenotype. Bioinformatic analysis revealed that B_IR_ cells had enhanced cell viability and epigenetic regulation, and that ALOX5AP, MIF, and PTPRC/CD45 expressed by myeloid cells may be critical coordinators of diverse biological processes of B_IR_ cells. Immunofluorescence confirmed the presence of B_IR_ cells in tertiary lymphoid structures (TLSs) in skin SCC, RCC, CRC, and breast cancer. B_IR_-associated gene signatures correlate with positive outcomes in patients with melanoma, glioblastoma, NSCLC, HNSCC, or RCC treated with ICI therapy, and B_IR_-cell density predicted NSCLC patients’ response to checkpoint immunotherapy. In line with this, melanoma-bearing mice depleted of B_IR_ cells were resistant to ICIs.

**Conclusions:**

CD20^+^CD22^+^ADAM28^+^ B_IR_ cells were present in cancer-associated TLS and promoted the response to ICI therapy.

## Introduction

Immunotherapy has afforded patients with melanoma and other cancers the potential for long-term survival. Considerable progress has been made in this regard, with the identification of several validated biomarkers, particularly for immune checkpoint inhibitor (ICI) therapy. It is clear that cytotoxic T cells have a dominant role in response to ICI and other forms of immunotherapy. However, there is a growing appreciation of different components of the tumor microenvironment that may influence the therapeutic response—including myeloid cells and other subsets of immune cells (1).

Tumor-infiltrating B cells have been identified, but their overall functional role in cancer is incompletely understood (2, 3). Some studies suggest that B cells are positively associated with improved cancer outcomes, particularly when they are localized in so-called tertiary lymphoid structures (TLSs), which have been identified in a wide range of cancers, including melanoma, sarcoma, and lung cancer (4). Chronic exposure to inflammatory signals induced TLS neogenesis in peripheral tissue (4). TLSs consist of T-cell-rich regions and germinal center (GC)-characterized B-cell follicles, which resemble secondary lymphoid organs (4). Their presence is associated with a favorable prognosis in most solid malignancies, including melanoma (5) and soft-tissue sarcoma (6).

Mature TLSs exhibit evidence for the formation of GC in cutaneous melanoma and non-small cell lung carcinoma (NSCLC) (7, 8). TLS neogenesis was divided into several sequential maturation stages, starting as dense lymphocytic aggregates and culminating in GC formation (7, 9). These GCs enrich with a cluster of B cells positive for CD20 (MS4A1), Ki67, activation-induced deaminase (AID), and BCL-6 (10, 11). Enrichment of switched memory B cells increases the prognosis of patients with ICIs (12). In addition, B cells can also present antigens to T cells, including CD8^+^ T cells. The engagement of CD80 and CD40 on B cells replaces the need for CD4^+^ T cells to activate CD8^+^ cytotoxic T cells in anti-tumor responses (11). The co-localization of both CD20^+^ B cells and CD8^+^ T cells correlates with increased patient survival in a large series of human cancers (11, 13). T cells in melanoma without TLSs had a dysfunctional molecular phenotype (5). Altogether, these studies demonstrate the supporting role of B cells in TLSs for T-cell activity.

Notably, although preliminary evidence suggests the indispensable role that B cells play in the TLS, the specific phenotype of TLS B cells remains unclear. Memory B cells in TLSs might quickly proliferate and differentiate into plasma cells, which secrete high-affinity antibodies to activate the humoral anti-tumor response under ICI treatment (12). In this study, we identify a new B-cell subpopulation [called ICI-Responsive B (B_IR_) cells] in the TLSs and consider it a potential prognostic marker in cancer immunotherapies. B_IR_ cells were identified as a subset of memory B cells that promoted the response to ICI therapy.

## Method

### Sequencing Datasets and Clinical Information

This study utilized single-cell RNA sequencing data from the following studies:

1. GSE120575, CD45^+^ cells of a melanoma anti-PD-1-treated cohort (14).
2. GSE123813, CD45^+^ and CD45^-^ cells of a basal cell carcinoma anti-PD-1-treated cohort (15).

The following bulk RNA sequencing datasets were also adopted:

1. GSE67501, a renal cell carcinoma anti-PD-1-treated cohort (16).
2. GSE78220, a melanoma anti-PD-1-treated cohort (17).
3. GSE91061, a melanoma anti-PD-1-treated cohort (18).
4. GSE93157, an anti-PD-1-treated cohort of melanoma, head and neck squamous cell carcinoma (HNSCC), and NSCLC (19).
5. GSE113126, a melanoma anti-PD-1-treated cohort (20).
6. GSE115821, a melanoma anti-PD-1/CTLA-4-treated cohort (21).
7. GSE121810, a glioblastoma anti-PD-1-treated cohort (22).
8. GSE126044, an NSCLC anti-PD-1/PD-L1-treated cohort (23).
9. GSE135222, an NSCLC anti-PD-1-treated cohort (24).
10. GSE136961, an NSCLC anti-PD-1-treated cohort (25).
11. PRJEB23709, a melanoma anti-PD-1/CTLA-4-treated cohort (26).
12. PRJEB25780, a gastric adenocarcinoma anti-PD-1-treated cohort (27).
13. syn21593960, a melanoma anti-PD-1-treated cohort (28).
14. The Cancer Genome Atlas (TCGA), 32 types of cancer histology, no ICI treated.

All clinical information was collected based on reference reports. A uniform clinical end-point response was defined based on radiological response as per the RECIST criteria, with ‘‘CR/PR’’ being classified as a responder and ‘‘SD/PD’’ being classified as a non-responder.

### Patient Data and Study Approval

FFPE surgery samples from 12 patients with NSCLC were obtained from the Wuhan Union Hospital according to IRB-approved protocols. All patients received surgery at Wuhan Union Hospital and experienced local recurrence or distant metastasis during postoperative follow-up. Then, they underwent anti-PD-1 treatment for recurrent unresectable or stage IV disease. Tumor samples were collected at baseline according to a standard pathology procedure. The response was defined as achieving a complete or partial radiographic response by iRECIST between pre-treatment imaging and post-treatment imaging (29). Target diseases, including lung and metastatic sites, were measured based on radiologic imaging, such as CT, MRI, and PET/CT.

### Preprocessing of scRNA-Seq Data

Single-cell RNA sequencing data were analyzed in the R statistical computing framework, version 4.0. The Seurat package was used to filter out bad-quality cells and normalize counts (30). Downstream analysis used log2-transformed normalized count data. All count data, metadata, and intermediate results were kept within a Seurat R object.

For all cells in scRNA-seq profiles, clusters were annotated based on the expression of known marker genes, including CD3G, CD3D, CD3E (T cells), CD8A (CD8^+^ T cells), CD4 (CD4^+^ T cells), CD45RO, SELL, CCR7 (memory T cells, including CD45RO^+^SELL^+^CCR7^+^ early memory T and CD45RO^+^SELL^-^CCR7^-^ late memory T), CD2, PDCD1, CTLA4 (exhausted T), GZMA (effector T cells), MKI67 (proliferative T cells), CD19, CD79A (B cells), SLAMF7, IGKC (plasma cells), FCGR2A, CSF1R (macrophages), CD14 (monocytes), CLEC4C (plasmacytoid dendritic cells), COL1A2, FAP, PDPN (fibroblasts), EPCAM, and TP63 (malignant cells). These annotations were also confirmed by identifying differentially expressed marker genes for each cluster and comparing them to known cell-type-specific marker genes. As for B cells, clusters were annotated based on the expression of multiple B-cell subpopulation markers, including CD10, CD19, CD20, CD22, CD24, CD27, CD38, and IgD (6). Then, we downloaded bulk RNA-seq count data from sorted immune cell populations from previously published studies and compared bulk gene expression to pseudo-bulk expression profiles from single-cell clusters. Unique molecular identifier (UMI) counts were summed for all cells in each cluster to generate pseudo-bulk profiles. Gene counts from aggregated single-cell and bulk data were then normalized and depth-corrected using variance stabilizing transformation in DESeq2 (version 1.18.1). Genes with a coefficient of variation >20% across bulk RNA-seq datasets were used to calculate the Pearson correlation between bulk datasets and pseudo-bulk profiles. Following the cell cluster annotation above, single cells from tumors were projected to 2D space using the UMAP with the color indication for cell types and patients’ responses to ICI therapy.

### Identification of B_IR_ Cells and Their Specific Gene Signature

In order to evaluate the clinical significance of each cell population, all cells were divided into two categories. Cells from patients who respond to ICI therapy were marked as “responsive” cells. Then, the ratio of “responsive” cells to all cells was calculated for each cell population. Among all cell clusters, the first B-cell cluster had the largest “responsive” cell ratios in both scRNA-seq profiles and thus was identified as B_IR_ cells.

Then, we explored differentially expressed genes among each cluster in scRNA-seq profiles by the function “FindAllMarkers” of the R package “Seurat” (30). The significant differentially expressed genes were defined as having FDR-adjusted *p*-value < 0.05 and |log2(fold change)| > 1. Clustered heatmaps of significant differentially expressed gene expression were generated with the R function “DoHeatmap” based on *z*-scores of log(TPM/10+1). The analysis for B_IR_ cell-specific gene signature was conducted as follows: (i) B_IR_ cell-specific genes discovered in two scRNA-seq datasets were filtered for *q* < 0.05 (FDR-corrected *p*-value), log10 fold-change > 2, and (ii) intersection of genes obtained from two datasets yielded *n* = 33 genes, which were then sorted based on the average fold change.

### B-Cell Function Analysis

Gene signatures of single cells were quantified by applying the gene set variation analysis (GSVA) (31) method with the R package “GSVA”, which calculated the signature enrichment scores of individual single cells independently without normalization across cells. The gene expression [log(TPM/10+1)] matrix was used, and single cells that did not express either GAPDH or ACTB were excluded from the analysis. We performed unbiased analysis on five gene ontology signatures (MSigDB, C5 sets), namely, “GO_B_CELL_ACTIVATION”, “GO_B_CELL_DIFFERENTIATION”, “GO_B_CELL_HOMEOSTASIS”, “GO_B_CELL_PROLIFERATION”, and “GO_B_CELL_RECEPTOR_SIGNALING_PATHWAY”. The resulting GSVA score matrix was organized as having single cells in the columns and the signatures in the rows. Comparisons of single-cell enrichment scores of two B cells were performed using the R package “limma” (32). Differentially enriched signatures were defined as having FDR-adjusted *p*-values < 0.05 and |mean score difference| ≥ 0.1.

### Trajectory Analysis

In the separate analysis of B cells, we extracted all B cells identified by cluster annotation above from the GSE120575 dataset and used the package “Monocle” to analyze the trajectory (33). As input to Monocle’s Reversed Graph Embedding algorithm, we selected a set of 426 genes that was the union of the top 100 differentially expressed genes ordered by ascending *q*-value for B-cell clusters. Single cells and related trajectory lines were projected to 2D space using the UMAP with the color indication for pseudotime, B_IR_ cells, and patients’ responses to ICI therapy.

### Ligand–Receptor Interactions

To illustrate the cell–cell communication potential of B_IR_ cells, we used CellPhoneDB (34) to predict enriched cellular interactions among all cell types from single-cell transcriptomics data. Both ligands and receptors expressed by B_IR_ cells were taken into account when calculating the means of the average expression level of two interacting molecules as the strength of each ligand–receptor pair. Fifty pairs with average largest strength were visualized in the dot plot. The sum of all significant ligand–receptor pairs was identified as the communication intensity between B_IR_ cells and every other cell population.

NicheNet is a method that predicts which ligands produced by one cell regulate the expression of which target genes in another cell (35). Ligand–target links were inferred by combining bulk or single-cell expression data of interacting cells with existing knowledge on signaling and gene regulatory networks. Ligand regulatory potential scores were calculated for each ligand–target pair, which indicated the ability of ligands to induce the expression of these genes from the B_IR_-cell signature. The top 7 ligands (out of 324 ligands) and top 7 targets from 33 genes of the B_IR_-cell signature were shown in the heatmaps. Ligands were ordered according to the average score over both settings.

### Gene Set Enrichment Analysis

To quantify the enrichment scores of the B_IR_-cell signature and TLS signature in bulk RNA-seq datasets, we applied the GSVA (31) and single-sample gene set enrichment analysis (ssGSEA) (36) method with the R package “GSVA” for each sample. Then, all samples were divided into two groups according to their enrichment scores, namely, the high-score (top 50%) and the low-score (bottom 50%) groups. A total of 14,765 gene ontology (GO) sets (MSigDB, C5 sets) were used to functionally annotate a 33-gene B_IR_-cell signature through the package “clusterProfiler”. Significant GO sets with adjusted *p*-values < 0.05 were shown in the dot plot. The function “goplot” visualized the graph about the relationship among enriched GO sets. The function “gseaplot” portrayed the enrichment of the B_IR_-cell signature in samples from patients’ responses to ICI therapy.

### Gene Network Analysis

The interaction between potentially targeted genes was performed using the Search Tool for the Retrieval of Interacting Genes (STRING; http://string-db.org) (version 11.0) online database (37). The cutoff value for STRING analysis was 0.01, considering only interactions curated from database or text-mining, experimentally determined, co-occurrent, and co-expressed. Analyzing the functional interactions between proteins might provide insights into the biological mechanisms of action. Cytoscape software was used to screen for the hub protein and build the regulation network.

### Prognosis Analysis

Cancer gene expression and clinical outcomes for each type of cancer were evaluated using PREdiction of Clinical Outcomes from Genomic Profiles (PRECOG) (38). *Z*-scores were obtained from the PRECOG website (http://precog.stanford.edu). Genes with negative PRECOG *z*-scores indicated significant favorable prognosis (filtered for |*z*-scores| > 3.09, or nominal one-sided *p* < 0.001). Global PRECOG meta-*z*-scores included 39 kinds of cancer histology, including breast cancer, melanoma, and NSCLC. The survival curves were plotted using the Kaplan–Meier method, using the log-rank test to compare the statistical differences between curves. The prognostic values of the B_IR_-cell signature were also evaluated using the multivariate Cox proportional hazards model, with AJCC cancer stage and pathology tumor grade as covariates. The analysis was done using the R package “survival”. All samples were separated into high versus low groups with the median value of gene expression or signature scores as the cutoff.

### Cell Culture and Small Interfering RNA Transfection

B16 cells (American Type Culture Collection) were cultured in RPMI1640 (Gibco) supplemented with 10% fetal bovine serum (Gibco) with penicillin/streptomycin (Gibco) at 37°C with 5% CO_2_. Splenic B cells were isolated with Magnetic Cell Sorting (MACS) beads by negative selection according to the manufacturer’s protocol (Miltenyi Biotec). The purity was routine >96% as assessed by staining with anti-CD19. For siRNA transfection, mouse Adam28 siRNA (Ribobio) or control siRNA (Ribobio) was used in the presence of riboFECT reagent (Ribobio). B cells were harvested 48 h later for qRT-PCR analysis. The following siRNAs were used: siAdam28-1: GCATGATTCATGACTACTT; siAdam28-2: GCAGTCGTGTCAATTACAA; and siAdam28-3: CACCAAGGATGCCAAGCTA.

### Animal Experiments

C57BL/6 mice (HFK Bioscience, Beijing, China) were bred in a specific pathogen-free facility, and female mice were utilized at 6–8 weeks of age. B16 tumor cells (5 × 10^5^ cells) were trypsinized, washed, and resuspended in PBS and injected subcutaneously (s.c.) in the flank of C57BL6 mice. Anti-PD-1 antibodies (BioXCell; clone RMP1-14) and siAdam28 (Ribobio) were individually injected intraperitoneally (i.p.) and intratumorally (i.t.) at the time points described in figure legends. For functional experiments, spleen, LNs (inguinal, axillary, and brachial), and tumors were dissected into RPMI. Mouse tumors were mechanically disrupted using scissors, digested with a mixture of 0.5 mg/ml DNase (Sigma-Aldrich) and 1 mg/ml Collagenase IV (Sigma-Aldrich) in serum-free RPMI for 30 min. The single-cell suspension of tumors was dispersed through a 70-µm filter. Erythrolysis of whole blood and spleen samples was performed using the BD Pharm Lyse buffer (BD). For tumor growth and control experiments, tumors were measured twice weekly and volumes were calculated as the product of three orthogonal diameters. Mice were sacrificed when any diameter reached 15 mm.

For flow cytometry analysis, samples were stained with Fixable Viability Stain 510 or 780 (BD Horizon) and fluorescent dye-conjugated antibodies anti-mouse CD45 (BD; HI30), CD3 (BD; SK7), CD4 (BioLegend; RPA-T4), CD8 (BioLegend; 53-6.7), CD19 (BD; 1D3), CD20 (BioLegend; SA275A11), CD22 (BioLegend; OX-97), CD25 (BD; M-A251), CD27 (BioLegend; LG.3A10), CD40 (BD; 3/23), IFNγ (BioLegend; XMG1.2), IgA (eBioscience; mA-6E1), IgD (BioLegend; 11-26c.2a), IL10 (BD; JES5-16E3), and ADAM28 (Santa Cruz; H-4). For FoxP3 detection, cells were stained using Transcription Factor Staining Set (BD) and anti-human FoxP3 antibody (236A/E7). For intracellular staining of IFNγ, Cytofix/Cytoperm solution (BD) was added prior to fixation and permeabilization. The acquisition was performed with FACS LSRII (BD Biosciences). Data analysis was performed in FlowJo v.0.5.3 (Tree Star). Flow cytometry graphs shown in the *Results* section were representative data from at least three independent experiments.

### Immunofluorescence

Immunofluorescence staining was performed on FFPE tumor tissue sections. The tumor tissues were fixed in 10% formalin, embedded in paraffin, and serially sectioned to 3 µm thick. The following primary antibodies were used: CD3 (Abcam, Clone CD3-12, dilution 1/400), CD20 (Abcam, Clone SP32, dilution 1/200), CD22 (Zsbio, Clone OTI4C3, no dilution), CD79b (Proteintech, 21063-1-AP, dilution 1/200), CD180 (Abcam, Clone EPR14720, dilution 1/200), and ADAM28 (Santa Cruz, Clone H-4, dilution 1/50). Double stainings of CD20 with CD3, CD22, CD79B, CD180, or ADAM28 were performed manually. Primary antibodies were detected with whole IgG or IgG F(Ab’)2 fragments conjugated to Alexa Flour 488 (711-546-152, Jackson ImmunoResearch) or streptavidin-conjugated Cy3 (016-160-084, Jackson ImmunoResearch). 4′,6-diamidino-2-phenylindole (DAPI) was used for visualization of cell nuclei (Sigma-Aldrich). For immunofluorescence multiplex staining, we followed the staining method for the following markers: CD20 with fluorescein FITC (1:50), CD22 with fluorescein Cy3 (1:50), ADAM28 with fluorescein Cy5 (1:50), and nuclei visualized with DAPI (1:2,000). A Nikon Ti-E microscope was used for all imaging. Image analysis was performed using NIS software modules (Nikon, version 4) and ImageJ. TLSs were qualified and quantified using both H&E and CD3/CD20 immunofluorescence staining. TLSs in the tumor area were identified as aggregates of lymphocytes having histological features with analogous structures to lymphoid tissue (follicles of CD20^+^ B cells surrounded by parafollicular zones of CD3^+^ T cells). For the current study, criteria used for the quantification of TLSs and B cells include the following: (1) the total number of structures identified either within the tumoral area or in direct contact with the tumor cells on the margin of the tumors (numbers of TLSs per mm^2^ area) (12), (2) the positivity of different markers in CD20^+^ B cells in TLSs (>5 TLSs were analyzed per marker), and (3) the percentage of CD20^+^CD22^+^ADAM28^+^ B cells in TLSs (>5 TLSs were analyzed per section). All material summarized in one graph was stained and imaged simultaneously with standardized threshold intensity. Quantification of TLSs and B_IR_ cells on immunofluorescence-stained tissues was independently performed by two trained pathologists who were blinded to the clinical and experimental data.

### qRT-PCR

Total RNA was extracted using TriZol Reagent (Invitrogen), and cDNA was generated using a HiFiScript^®^ cDNA Synthesis kit (CW Biotech, Beijing, China). qRT-PCR analyses were carried out using an SYBR Green Real-time PCR kit (Toyobo, Osaka, Japan) in a LightCycler^®^ (Bio-Rad Laboratories, Hercules, CA, USA). The expression of individual genes was calculated by a standard curve method and normalized to the expression of GAPDH. Fold changes were analyzed using the formula: 2^−ΔΔCt^. Gene expression was detected using the following primers: NM_010082-F: AGCCTCCACCTGATGTCCTAA, NM_010082-R: AGGTACACGCGGCCTATTTG, NM_183366-F: AGCCTCCACCTGATGTCCTAA, NM_183366-R: AGGTTAGCCTAGGGAGCACT, GAPDH-F: CGGGAAGCTCACTGGCATGGC, and GAPDH-R: GGTGGAGGAGTGGGTGTCGCTGTT.

### Statistical Methods

Unless otherwise stated, Mann–Whitney *U* test was used to assess for a difference in distributions between two population groups. Statistical analyses were carried out using R4.0.1 (http://www.r-project.org/) or greater. We considered *p*-value < 0.05 as being statistically significant.

## Result

### Immune Cells in the Tumors of ICI Responders

To gain insight into the potential functional role of different kinds of cells in response to ICI, we analyzed two single-cell RNA sequencing (scRNA-seq) profiles from tumor tissues of melanoma (GSE120575) and basal cell carcinoma (BCC; GSE123813) (14, 15). Patients in both studies were treated with ICI therapy (with anti-PD-1 or anti-CTLA4) and were divided as responders (17 melanoma and 6 BCC) and non-responders (31 melanoma and 5 BCC) according to RECIST criteria.

**Figure 1:**
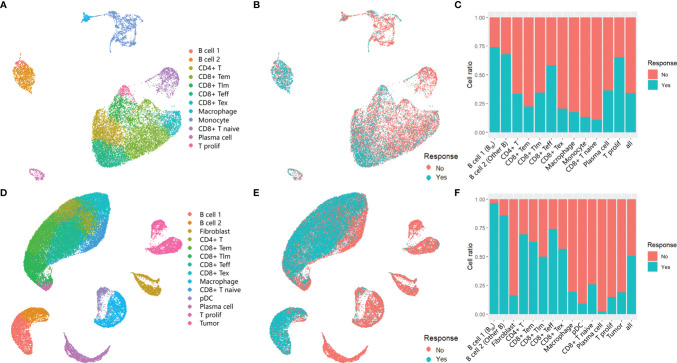
Immune cells in the tumors of ICI responders. **(A, B, D, E)** UMAP plot of all cells in the tumors of patients treated with ICI,which visualize cell expression profiles in a two-dimensional. independent space. Data from two scRNA-seq datasets GSE120575 **(A, B)** and GSE123813 **(D, E)** were analyzed. Cells are colored based on clusters defined by cell type **(A, D)** and patients' response to ICItherapy **(B, E)**. Tem, early memory T cells; Tim, late memory T cells;Teff, effector T cells;Tex, exhausted T cells;T prolif, proliferative T cells;pDC, plasmacytoid dendritic cells. **(C, F)** Histogram of the ratio of cell numbers from patients who respond to ICI therapy compared wi th all patients. Each cell type in profiles GSE120575 **(C)** and GSE123813 **(F)** are concerned.In the following text,'B cell1' is named as 'ICI-Responsive B cells (B_IR_)' because of its highest cell ratio in tumor tissue of patients with favorable response to ICI therapy. Correspondingly, 'B cell 2' is called 'other B cells'.

Based on the same analysis workflow, the Seurat clustering of these cells identified 15 distinct major clusters representing epithelial, immune, endothelial, and fibroblast populations. Next, we employed lineage-specific markers to annotate clusters into major cell types of the tumor, including T cells (CD3G, CD4, CD8A, CD28, and IL7R), B cells (CD19 and CD20), myeloid cells (HLA-DRA and CD14), fibroblasts (THY1 and ACTA2), and tumor cells (EPCAM and S100A) (**Figures S1A, B**). In total, we identified seven T-cell clusters, three myeloid clusters, two B-cell clusters, two stromal clusters, and a tumor cell cluster (**Figures 1A, D**). No stromal and malignant cells were identified in GSE120575 because only CD45^+^ cells were sorted for sequencing. Cells from responders and non-responders to ICI therapy were distributed in different cell clusters (**Figures 1B, E**). Two B-cell clusters and CD8^+^ Teff (effector T) cell clusters were more frequent in responder lesions, while macrophage and CD8^+^ Tex (exhausted T) cell clusters were more frequent in non-responder lesions in both datasets (**Figures 1C, F**). The first B-cell cluster (B cell 1) indicated the best response to ICI therapy among all cell clusters in both datasets, suggesting that a B-cell subset, which we called B_IR_ below, played a significant role in the efficacy of checkpoint therapies.

### Phenotype of ICI-Responsive B Cells

**Figure 2:**
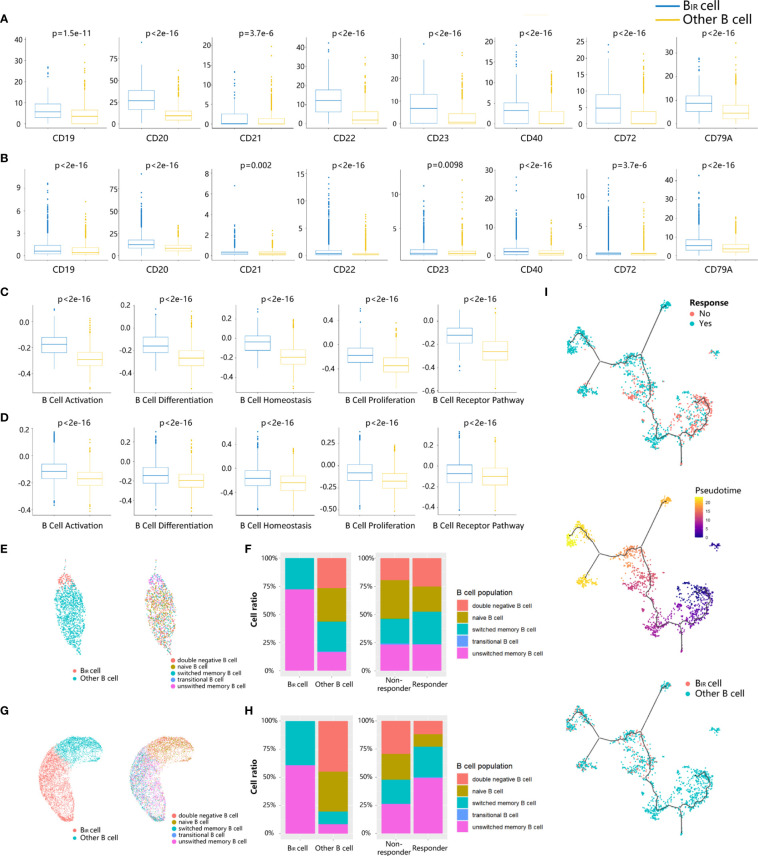
Phenotypes of B_IR_ cells. **(A, B)** Bar graphs show that seven B cell marker genes (CD19,CD20, CD21, CD22,CD23, CD40, CD72,CD79A) are more significantly. (p <0.05) upregulated in B_IR_ cells than in other B cells from profiles GSE120575 **(A)** and GSE123813 **(B)**. scRNA-sequencing data are normal ized as transcripts per million (TPM). The expression is displayed as the log2(TPM+1) of each cell from two clusters. **(C, D)** Bar graphs show that five B cell-related gene sets (activation, differentiation, homeostasis,proliferation, BCR pathway) are more significantly (p <0.05) enriched by B_IR_ cells than by other B cell populations from profiles GSE120575 **(C)** and GSE123813 **(D)**. The enrichment is displayed as the gene set variation analysis (GSVA) scores of each cell from two clusters. **(E, G)** UMAP plots of B cells in the tumors of patients treated with ICI from two scRNA-seq datasets GSE120575 **(E)** and GSE123813 **(G)**. Cells are colored based on two B cell populations including B_IR_ and other B cells (left) and five B cell subpopulations defined by biomarkers (right). **(F, H)** Histogram of frequency of B cell subpopulations from profiles GSE120575 **(F)** and GSE123813 **(H)**. Cells are grouped based on B cell populations including B_IR_ and other B cells (left) and patients' response to ICI therapy (right). **(I)** Trajectory analysis for the B cells.B cells from GSE120575 are reclustered and visualized by UMAP plots. The solid black line indicates the main diameter path of UMAP and provides the backbone of Monocle's pseudotime ordering of the cells, indicating B cell differential states. Each dot represents an individual cell colored by patients' response to ICI therapy (up), by pseudotime (middle) or by cluster (down).

To explore the phenotype of B_IR_ cells, we first applied differential gene expression analysis, which could reveal the increased expression of BCR coreceptors (CD19, CR2/CD21, and CD79A), B-cell activation genes (CD20 and CD40), B-cell inhibitory genes (CD22), and B-cell differentiation regulation (CD23, CD27, and CD72) in B_IR_ cells versus other B cells (**Figures 2A, B**). B_IR_ cells had higher levels of B-cell activation, differentiation, proliferation, homeostasis, and BCR pathway (**Figures 2C, D**). Overall, B_IR_ cells tend to initiate stronger immune responses than other B cells.

Then, we assessed the functional subpopulation of B_IR_ cells. Multiple B-cell subpopulation markers are unevenly expressed in B cells, such as CD22, CD27, and CD38, suggesting B-cell heterogeneity in the tumor microenvironment (**Figures S2A, B**). Based on these markers, B-cell populations were divided as naive (CD19^+^CD27^−^IgD^+^), transitional (CD19^+^CD24^++^CD38^++^CD10^+^CD27^−^IgD^+^), unswitched memory (CD19^+^CD27^+^IgD^+^), switched memory (CD19^+^CD27^+^IgD^−^), and double-negative (CD19^+^CD27^−^IgD^−^) populations in a supervised manner (**Figures 2E, G**, **S2C, S2D**) (6). B_IR_ cells mainly comprised unswitched and switched memory B cells in both scRNA-seq profiles (**Figures 2F, H**). We next compared the phenotypes of B cells in tumors from responders and non-responders to ICI treatment. Tumors from responders had moderately more memory B cells and significantly less naive B cells, which was consistent with previous literature reports (**Figures 2F, H**) (6). In conclusion, B_IR_ cells were a cluster of memory B cells that contributed to patients’ response to ICI therapy.

Since B cells were in continuous state transition in cancers, we used trajectory analysis (33) for the B-cell population in melanoma scRNA-seq profile to identify the main trajectory branch and three side branches, reflecting a possible path for B-cell differentiation from less pseudotime to more pseudotime (**Figure 2I**). B cells from non-responders accumulated in the clusters with less pseudotime, indicating naive or immature B cell states, while responders had a significantly higher frequency of mature B cells with more pseudotime (**Figure 2I**). Similarly, B_IR_ cells also appeared to keep a mature state, which was related to patients’ response to ICI therapy.

### Communication Between B_IR_ Cells and Other Cells

**Figure 3:**
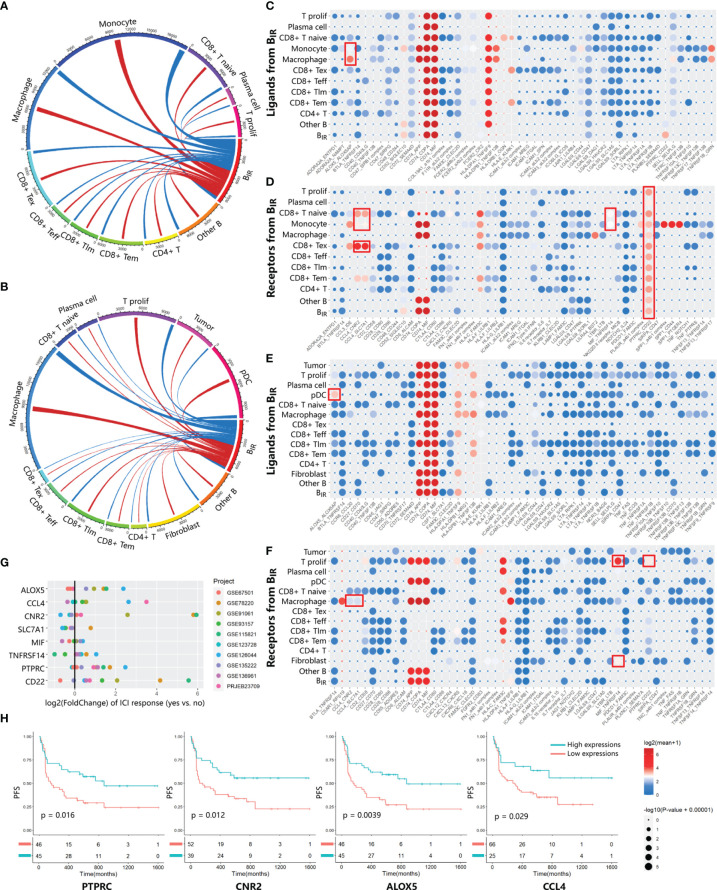
Communication between B_IR_ cells and other cells. **(A, B)** Chord diagrams of cellular interactome between B_IR_ cells and all other cell clusters from two tumor single-cell atlas GSE120575 **(A)** and GSE123813 **(B)**.The number of interactions among clusters are indicated by line thickness.Interactions between ligands in B_IR_ cells and receptors in other clusters are marked in red. Interactions between receptors in B_IR_ cells and ligands in other clusters are marked in blue. **(C–F)** Overview of significant ligand-receptor interactions from data sets GSE120575 **(C, D)** and GSE123813 **(E, F)**. Ligands **(C, E)** and receptors **(D, F)** expressed by B_IR_ cells interact with relevant molecules of all other clusters. Ligand-receptor pairs with strong interaction are portrayed as circles with low P values (large circles) and high means of the average expression level of two interacting molecules (red circles). Meaningful interactions in both data sets are marked by red rectangles.**(G, H)** Evaluation of the prognostic significance of genes from meaningful ligand-receptor interactions.**(G)** The fold change of gene expression in ICIresponders as compared to non-responders is calculated for eight genes from meaningful ligand-receptor interactions.10 datasets from patients treated with ICI therapy are indicated by colors. Only significant fold change (p<0.05) are showed in the plot. **(H)** Correlations of four genes in PRJEB23709 with progression-free survival(PFS). The red line designates the samples with lowly expressed genes,and the blue line indicates the samples with highly expressed genes.

To investigate the role of B_IR_ cells in the immune microenvironment, we used CellPhoneDB (34) to predict specific or enriched receptor–ligand interactions between B_IR_ cells and other cell clusters. **Figures 3A, B** show that both ligands and receptors expressed on B_IR_ cells are mostly bound to macrophages in two scRNA-seq profiles. B_IR_ cells also strongly interacted with subpopulations of myeloid cells (monocytes and pDC) and T cells (CD8+ Tex, CD8+ T naive, and T prolif) in individual cohorts. We identified statistically significant strong interactions for ALOX5AP-ALOX5, CCL4-CNR2/SLC7A1, MIF-TNFRSF14, and CD22-PTPRC, which were likely to be important in B_IR_-cell function (**Figures 3C–F**). Notably, B_IR_ cells expressed high levels of MIF, the receptor of which (CD74) was expressed by macrophages, monocytes, and B cells. As a pivotal regulator of innate immunity, MIF secreted by B cells induced inflammatory responses and prevented activation-induced apoptosis in myeloid cells (39). In addition, MIF directly promoted B-cell migration and proliferation through non-cognate interaction with CD74 (40). Therefore, MIF expressed by B_IR_ cells might affect both myeloid cells and B cells themselves. Interaction between CD45 and CD22 promoted CD22 organization in nanodomains and limited the association of CD22 to the BCR in resting B cells, which promoted BCR signaling (41). These findings implied that the BCR signaling of B_IR_ cells could be promoted by CD45 on other immune cells. Among these interaction molecules, ALOX5, CCL4, CNR2, PTPRC, and CD22 were expressed more in ICI responders than in non-responders (**Figure 3G**). Furthermore, high expression of these genes also correlated significantly with improved progression-free survival (**Figure 3H**). In conclusion, some interaction between B_IR_ cells and other cells might play a considerable role in the prognosis of ICI therapy.

### B_IR_ Cell-Specific Gene Signature

**Figure 4:**
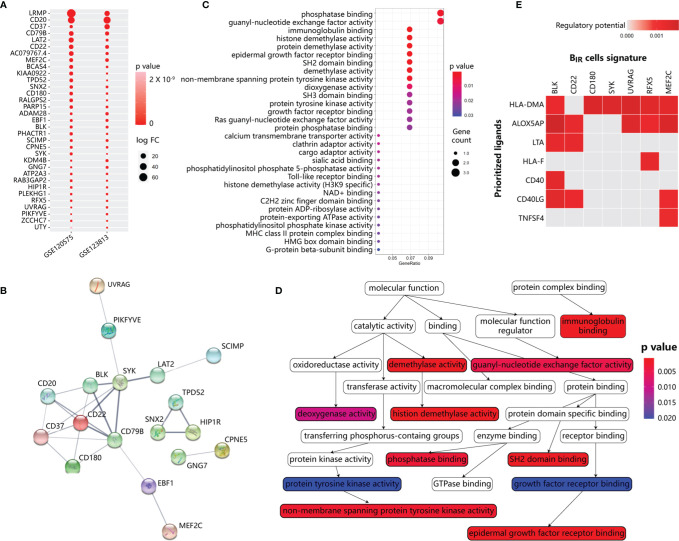
Functional annotation and network analysis of B_IR_ cells signature. **(A)** Specific gene signature of B_IR_ cells compared with other clusters. 33 genes are specifically expressed in B_IR_ cells in both scRNA-seq data sets. P values are indicated by color. The average fold change in expression of each gene in B_IR_ cells compared with other clusters are indicated by circle size.**(B)** Protein-protein interaction (PPI) network diagram for 33 genes among B_IR_ cells signature. The colorful network nodes and the lines represent proteins and protein-protein associations, respectively. The line thickness reflects the PPI strength between each protein pairs. **(C)** Significant gene sets enriched in B_IR_ cells signature. For each gene set, the gene counts enriched in this gene set are indicated by circle size, and p value for the enrichment of this gene set is indicated by color.**(D)** Relationship of gene sets enriched in B_IR_ cells signature. Each gene set points to its subsets with an arrow, and significant gene sets are marked with p value-de­ pendent color.**(E)** NicheNet analysis of upstream ligand-target pairs inducing genes of B_IR_ cells signature. Regulatory potential scores are calculated for each ligand-target pair, which indicates the ability of ligands to induce the expression of these genes from ICI-response B cells signature. In the heatmaps, the top 7 ligands (out of 324 1igands) and top 7 targets from 33 genes of B_IR_ cells signature are shown.

To identify B_IR_-cell programs, we used scRNA-seq profiles to define cell-type-specific expression signatures of B_IR_ cells, among the genes at a false discovery rate (FDR) of <1% differentially expressed when compared with other clusters (**Figures S3A, B**). To avoid mRNA contamination by tumors and other immune cells, we restricted the B_IR_-cell signatures only to a few hundred genes that were specifically expressed by B_IR_ cells but not other cells. Then, we intersected the B_IR_-cell signatures from the two scRNA-seq datasets, resulting in the discovery of 33 specific genes (**Figure 4A**). Consistent with a previous report, B_IR_ cells were significantly enriched with known unique signatures of B cells, such as CD20, CD22, CD37, CD79b, CD180, and BLK (13, 42). Markers like ADAM28, LAT2, LRMP, MEF2C, PARP15, PHACTR1, and SYK were also confirmed to be expressed at high levels in pre- or mature B cells (13, 43). However, RFX5, SNX2, and ZCCHC7 were recognized to have low tissue specificity and immune cell specificity before (43). In order to further find the hub genes of the B_IR_-cell signature in the network, STRING database (http://string-db.org) was applied. The results showed that 11 genes are related to each other, and the top hub genes were CD22, CD79B, SYK, BLK, CD20, CD37, and CD180, which overlapped with known unique signatures of B cells (**Figure 4B**). Therefore, a specific gene signature is identified to define B_IR_ cells in the tumor.

Based on gene set enrichment analysis (GSEA) using the ontology gene sets from the Molecular Signatures Database, the B_IR_-cell signature mostly associated with phosphatase binding and guanyl-nucleotide exchange factor activity (**Figures 4C, D**). Therefore, B_IR_-cell activity could be dependent on the phosphorylation status of protein and GTP. In addition, B_IR_ cells were enriched in histone demethylation activity, which was essential for B-cell activation and proliferation but not differentiation in response to BCR or TLR stimulation (44, 45). In summary, B_IR_ cells had several critical features, including protein phosphorylation and histone demethylation.

Then, we explored major ligands that affected the expression of B_IR_-cell signatures (**Figure 4E**). To do so, we used NicheNet, an algorithm that inferred ligand–receptor interactions inducing gene expression variations by combining transcriptome data of interacting cells with existing knowledge on signaling and gene regulatory networks (35). We applied NicheNet to predict which ligands could potentially induce expressions of B_IR_-cell signatures (**Figure 4E**). The top 7 predicted ligands were HLA-DMA, ALOX5AP, LTA, HLA-F, CD40, CD40LG, and TNFSF4. Notably, ALOX5AP, which significantly promoted downstream expressions of most genes of B_IR_-cell signatures, was also described as a key signaling molecule released by myeloid cells (**Figures 3C, E**). In activated immune cells, ALOX5AP acted as a scaffold that governed the distribution of 5-lipoxygenase (5-LOX) to the perinuclear region, increased the synthesis of the efficient leukotrienes, and consequently increased antibody production (46, 47). Therefore, we speculated that myeloid cells secreted ALOX5AP to activate B_IR_ cells.

### B_IR_ Cells in Predicting Prognosis

**Figure 5:**
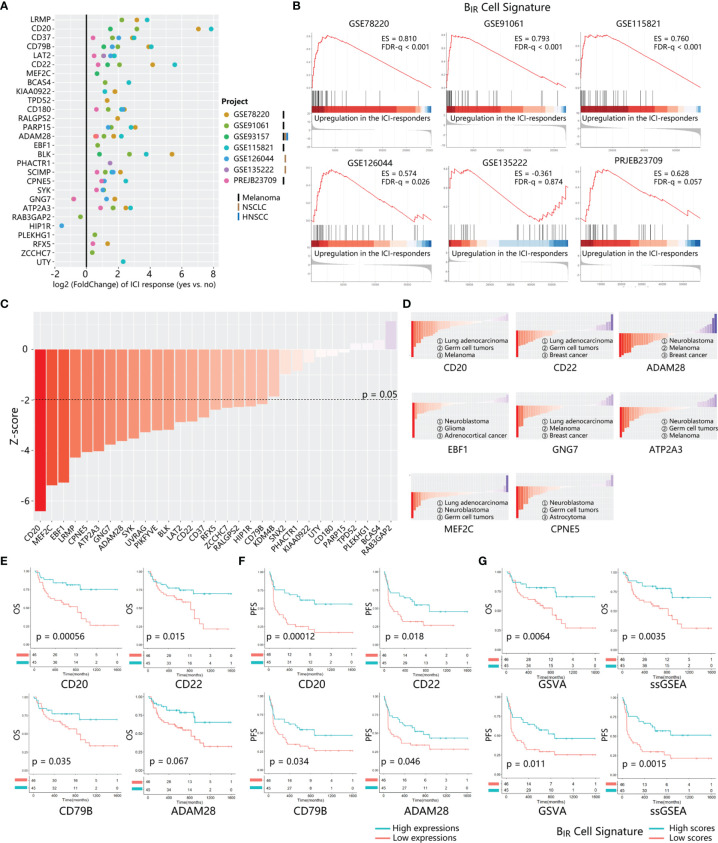
Prognosis value of B_IR_ cells signature in ICI-treated patients. **(A)** The fold change of gene expression in ICI responders as compared to non-responders is calculated for 28 genes from B_IR_ cells. signature. 7 transcriptome datasets from ICI-treated patients with different tumor types (marked by different bars) are indicated by circle colors. Only significant fold changes (p < 0.05) are shown in the plot. **(B)** GSEA for gene expression profiles of B_IR_ cells signature in multiple data sets. Hits (shown as black lines) are the transcripts of B_IR_ cells signature showing upregulation in the ICI-responders compared with non-responders. Enrichment scores (ES) and false discovery rate (FOR) q value are shown beside enrichment plots. **(C)** Global PRECOG z-scores of 31 genes from B_IR_ cells signature reflect their pan-cancer prognostic value. Genes are sorted according toz-score from favorable (red) to adverse prognostic genes (blue) in all kinds of cancers histology. The z-score threshold is set as ±1.96 (two-tailed P < 0.05) **(D)** PRECOG z-scores of each gene in different kinds of cancers histology.Histology with negative and positive z-scores individually indicates favorable (red) or adverse (blue) prognostic value of the gene in this tumor type. Solid tumor types with the top 3 minimum z-scores are shown for each gene. Correlations of critical genes of B_IR_ cells signatures with overall survival (OS) **(E)** and progression-free survival (PFS) **(F)** in PRJEB23709.The red line designates the samples with lowly expressed genes, and the blue line indicates the samples with highly expressed genes. **(G)** OS (up) and PFS (down) analysis of B_IR_ cells signature scores in PRJEB23709. Gene signature scores are quantified by GSVA (left) or ssGSEA (right) method. The red line designates the samples with lowly B_IR_ cells signature scores, and the blue l ine indicates the samples with highly B_IR_ cells signature scores.

To evaluate the prediction effect of the B_IR_-cell signature, we calculated the fold change of gene expression in responders compared to non-responders in several bulk RNA-seq profiles. Altogether, 8 datasets with patients’ responsive information were used. A total of 333 patients were diagnosed with melanoma, non-small cell lung carcinoma (NSCLC), or renal cell carcinoma (RCC) and treated with anti-PD-1, anti-PD-L1, or anti-CTLA-4 therapy. Overexpression of most genes in the B_IR_-cell signature indicated a good prognosis in ICI therapy (**Figure 5A**). Known B-cell signatures, such as CD20 (MS4A1), CD22, CD79b, and BLK, exhibited excellent predictive ability. Gene sets integrated from the B_IR_-cell signature were significantly enriched in responders during ICI therapy in almost all bulk RNA-seq profiles (**Figure 5B**). In conclusion, specific gene signatures extracted from B_IR_ cells predicted a good prognosis in ICI therapy.

To further explore a cancer-wide map of B_IR_ cells as biomarkers of all patients’ clinical outcomes, we used PRECOG to evaluate the prognostic power of each signature gene (33). Twenty and 0 genes among the whole B_IR_-cell signature individually predicted favorable and adverse prognosis in pan-cancer patients (filtered for |*z*-scores| > 1.96, or nominal two-tailed *p* < 0.05) (**Figure 5C**). With respect to melanoma and NSCLC patients, most genes in the B_IR_-cell signature also indicated a favorable prognosis (**Figure S4A**). Then, we further analyzed the prognostic prediction ability of these genes in each cancer histology. Some genes consistently indicated a positive prognosis in different cancer types, such as CD20, ADAM28, MEF2C, CPNE5, and ATP2A3 (**Figure 5D** and **Table S2**). On the contrary, EBF1 (*z* = −11.34) and GNG7 (*z* = −5.813) were the brain neuroblastoma- and lung adenocarcinoma-specific positive prognostic genes, respectively (**Figure 5D** and **Table S2**).

Then, survival analyses were performed to compare the B_IR_-cell signature of patients in TCGA datasets, including patients with different cancer histology who did not receive ICI therapy. Among 32 types of cancer histology, patients with high B_IR_ cells had a significant favorable overall survival in 6 types, including ACC (adrenocortical carcinoma, *p* = 0.0057), HNSC (head and neck squamous cell carcinoma, *p* = 0.00043), KIRC (kidney renal clear cell carcinoma, *p* = 0.031), LIHC (liver hepatocellular carcinoma, *p* = 0.0089), SARC (sarcoma, *p* = 0.036), and SKCM (skin cutaneous melanoma, *p* = 0.0039) (**Figure S5**).

The prognostic value of this B-cell signature in ICI-treated patients was validated by several bulk RNA-seq profiles. The results of the survival analysis suggested that high expressions of CD20, CD22, CD79B, ADAM28, BLK, and RFX5 correlated with improved overall survival (**Figures 5E**, **S4B**). In addition, high expressions of CD20, CD22, CD79B, ADAM28, BLK, PARP15, PHACTR1, and ZCCHC7 correlated significantly with improved progression-free survival (*p*-value < 0.05) (**Figures 5F**, **S4B**). Hence, combined with the results of PRECOG and survival analysis, it implied that the high expressions of CD20, CD22, CD79B, and ADAM28 might predict a positive response to anti-PD-1 immunotherapy for pan-cancer patients. To evaluate the impact of whole gene signatures of B_IR_ cells on prognosis, we integrated all individual genes as a single gene set, scored the enrichment degree of gene set by ssGSEA and GSVA methods, and divided patients into those with high and low scores. Survival analysis showed that patients with high scores for this B-cell signature had prolonged overall survival and progression-free survival (**Figure 5G**). With respect to melanoma and NSCLC patients integrated from multiple GEO datasets, patients with higher B_IR_-cell signature scores had longer progression-free survival (**Figure S4C**). In addition, melanoma and NSCLC patients who are responsive to ICI therapy had higher B_IR_-cell signature scores (**Figures S4D, E**). Thus, B_IR_ cells had a favorable impact on the clinical outcomes of patients with immunotherapy.

### B_IR_ Cells Accumulate in Tertiary Lymphoid Structures

It was reported that B cells within TLSs played a significant role in response to ICI in patients with metastatic melanoma and RCC (38). To explore the relationship between B_IR_ cells and TLS formation, we used a 42-gene signature for the detection of TLSs identified from transcriptomic analysis of human cancers, which included chemokine (CCL2, CCL3, CCL4, CCL5, CCL11, CCL14, CCL18, CCL19, CCL20, CCL21, CCL22, CXCL9, CXCL10, CXCL11, CXCL13, CCR1, CCR3, CCR5, CCR7, and CXCR3) and immune cell (BST1, CD4, CD5, CD6, CD38, CD40, CD200, CD274, CSF2, GFI1, ICAM1, ICOS, IGSF6, IL1R2, IL1RN, IL2RA, IRF4, PDCD1, SH2D1A, STAT5A, TIGIT, and TNFRSF17) signatures (4). A favorable impact of TLS density on the prognosis of patients had been observed irrespective of the detection method in multiple cancers (4). Overexpression of most genes in the TLS signature indicated a good prognosis in ICI therapy (**Figure S6A**). Then, we performed survival analysis for individual genes and found that almost half (18/35) of TLS signature genes correlated with prolonged survival (**Figures S6B–D**). The high ssGSEA and GSVA scores for TLS signatures also predicted better survival (**Figure S6E**). Therefore, TLS signatures were consistent with the B_IR_-cell signature as the favorable prognosis markers.

**Figure 6:**
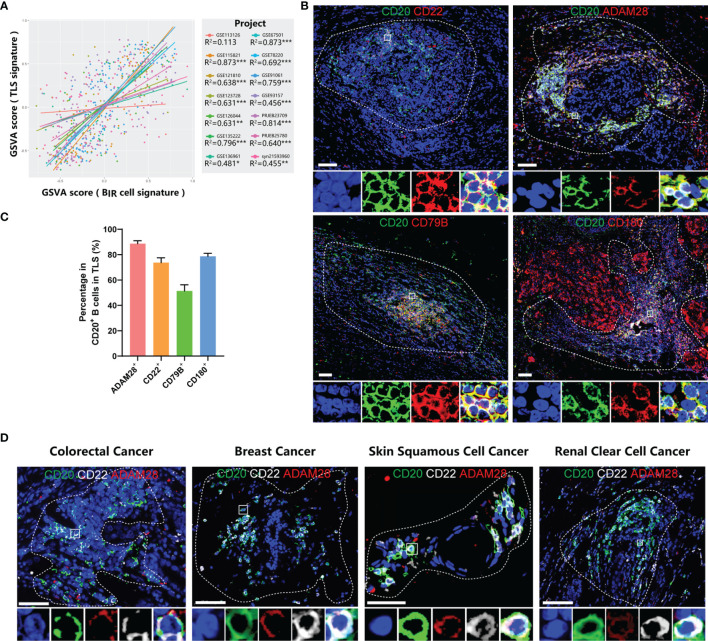
B_IR_ cells accumulate in TLSs. **(A)** Linear regression analysis between GSVA scores of B_IR_ cells signatures and TLSs. Samples (dots) wi th a higher GSVA score indicate a higher degree of enrichment of this signature. Samples (dots) and trendlines (lines) in different data sets are drawn with different colors. Correlation coefficient are presented as R2. *P < 0.05; **P < 0.01; ***P < 0.001. **(B)** Immunofluorescence staining of B_IR_ cells in breast cancer for CD20 (green) and the indicated markers (red), including ADAM28, CD22 , CD79B, and CD180. **(C)** Percentage of posi tive cells for these marker in CD20+ B cells in TLSs. **(D)** Characterization of B_IR_ cells for CD20 (green),CD22 (white), and ADAM28 (red) in colorectal cancer, breast cancer, skin squamous cell cancer and renal clear cell cancer. **(B, D)** The area of TLS is indicated by white dashed l ines based on related immunofluorescence staining for TLS in supplementary figure 7. The multispectral image of B_IR_ cells in the white rectangle is displayed below the original picture.All DAPI staining is shown in blue.Scale bar= 50m.

Using GSVA scores to evaluate enrichment of gene sets, we found that B_IR_ cells and TLS presence were significantly positively correlated in 13/14 bulk RNA-seq profiles, although there was no overlapping gene between these two signatures (**Figure 6A**). Consistent with the overall positive correlation, most individual genes in B_IR_-cell signatures predicted TLS presence in cancers (**Figure S7A**). Notably, 20/32 (62.5%) genes of the B_IR_-cell signature, such as CD20, CD22, and ADAM28, had a high correlation coefficient with TLS presence (**Figures 4A**, **S7A**). In conclusion, increased B_IR_ cells indicated high TLS abundance in cancer tissues.

On the basis of the results from gene expression profiling, we next assessed tumor samples histologically to gain insight into the distribution of B_IR_ cells as well as their relationship to TLSs in cancer patients. Consistent with a series of milestone studies, we detected TLSs by immunofluorescence double staining showing CD20^+^ B-cell zones and CD3^+^ T-cell zones (**Figures S7B–D**) (4, 12, 48). To explore markers for B_IR_ cells in TLSs, we took the intersection of genes with a high correlation coefficient for TLS presence and genes with a favorable prognosis for ICI-treated patients. Then, we excluded 2 genes that had low cell specificity before and were not suitable as markers. In this way, we filtered 5 candidate genes, including CD20, CD22, CD79B, CD180, and ADAM28. Next, we performed immunofluorescence staining of B cells in TLS to see whether they expressed candidate genes. ADAM28, CD22, and CD180 had a positivity of 88.4%, 73.6%, and 78.7% in CD20^+^ B cells in TLSs, respectively, while only 51.3% of B cells were CD79B^+^ (**Figure 6C**). CD180 was not uniquely expressed by B cells but was also expressed in tumor tissues (**Figures 6B**, **S7B**). Thus, we defined B_IR_ cells as CD20^+^CD22^+^ADAM28^+^ B cells. The presence of CD20^+^CD22^+^ADAM28^+^ B cells in the TLSs was verified in multiple types of cancers, including colorectal cancer (CRC), breast cancer, skin squamous cell cancer (SCC), and renal clear cell cancer (RCC) (**Figures 6D**, **S7D**).

It remained unclear whether B_IR_ cells were also present in normal secondary lymphoid organs (SLOs) in addition to the TLSs. Therefore, we evaluate ADAM28 expression in the SLOs to imply the possibility of B_IR_-cell localization. First, the human protein atlas showed that ADAM28 is not detected in the B cells of the appendix, spleen, tonsil, and lymph nodes (**Figure S8A**) (43). Second, we performed immunohistochemistry of human tonsil tissues with the same anti-ADAM28 antibody used in TLS staining. ADAM28 is not expressed in the germinal center of the tonsil (**Figure S8B**). In total, ADAM28 is not expressed in the B cells in SLOs, inferring that ADAM28^+^ B_IR_ cells are present only in TLSs but not in SLOs.

### B_IR_ Cells Mediate Immunity in ICI Therapy

**Figure 7:**
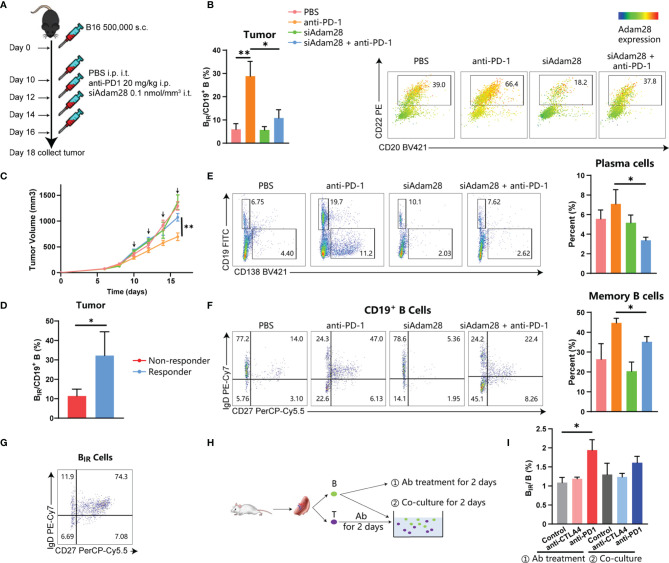
Active role for B_IR_ cells in ICI therapy. **(A–G)** C57BL6 mice were injected with 500,000 B16 tumor cells. Once tumors were palpable, mice were injected intraperitoneally (i.p.) with anti-PD1 (20 mg/kg) and/or intratumorally (i.t.) with siAdam28 (0.01 nmol/mm3) on days 10, 12,14 and 16.Tumors were collected on day24 after tumor inoculation and processed as described in Methods **(A)**. Each group was marked by a different color **(B. C, F)** and the mean tumor volume of the tumor-bearing mice was measured **(C)**, n=6 mice/group (only 12 mice for the anti-PD1 group). Data are presented as mean values ±SEM. **(B)** Flow cytometry analysis of CD20+CD22+Adam28+ B_IR_ cells in tumor tissues. The heatmap shows a projection of Adam28 expression onto all tumor CD45+CD19+ B cells and the histogram shows the percentage of B_IR_ cells in all CD19+ B cells. **(D)** Percentage of B_IR_ cells in all CD19+ B cells in tumor tissues were compared between mice that respond (n=S) or not respond (n=7) to ICI therapy. **(E)** Determination (left) and quantification (right) of plasma cells (CD19-CD138+) in CD45+ immune cells of tumor tissues. Determination of memory subsets of CD19+ B cells **(F)** and B_IR_ cells **(G)** i n tumor tissues. **(H, I)** B cells and T cells were separated from the spleen of the mouse. CD B cells were directly treated with anti-PD1 (20g/m)lor anti-CTLA4 antibodies (201-Jg/ml);@ T cells were pretreated with these antibodies and then co-cultured with B cells.The percent of B_IR_ cells in all B cells was quantified **(I)**.Data are mean values ±SEM, and individual data points are shown. *P < 0.05; **P < O.D1.

To establish the causative relationship between B_IR_ cells and ICI therapy, we tried to deplete Adam28^+^ B_IR_ cells *in vivo* through RNA interference for Adam28, because neutralizing antibodies or antagonists were not available. Three kinds of siAdam28 (siRNA for Adam28) were designed and validated through qPCR, while the first one was the most effective in inhibiting Adam28 expression in B cells and was picked out for subsequent animal experiments (**Figure S9A**). C57BL6 mice were injected with mouse melanoma cell lines and treated with anti-PD-1 antibody as ICI therapy and/or siAdam28 (**Figure 7A**). ICI therapy significantly increased B_IR_-cell infiltration in the tumor, lymph node, and spleen (**Figures 7B**, **S9B**). Compared with ICI-treated mice, siAdam28 intratumoral injections were confirmed to deplete B_IR_ cells in both tumor and draining lymph nodes but not blood or spleen (**Figures 7B** and **S8B**), suggesting that tumor-infiltrating B_IR_ cells were accurately depleted through siAdam28 treatment. Then, we found that B_IR_-cell depletion inhibited melanoma response to anti-PD-1 antibody (**Figure 7C**). All mice were divided into responders and non-responders based on tumor volume evaluation through RECIST criteria. B_IR_ cells were enriched in the tumor microenvironment of responders to ICI therapy (**Figures 7D**, **S9C**). B_IR_-cell depletion also decreased the percentage of memory B cells and plasma cells in the tumor (**Figures 7E, F**). Consistent with the result of bioinformatic analyses above, most B_IR_ cells belonged to unswitched memory (CD19^+^CD27^+^IgD^+^) (**Figure 7G**). Therefore, we speculated that B_IR_ cells functioned as memory B cells, which differentiate into plasma cells. In addition, B_IR_-cell depletion promotes IgA expression in both B cells and plasma cells (**Figure S9D**). IgA^+^ plasmocytes within prostate tumors were reported to diminish tumoral CTL activation through TGFβ receptor signaling (4, 12, 49). Consistently, we deduced that B_IR_ cells declined to differentiate into immunosuppressive IgA^+^ plasma cells, which inhibited Treg production (**Figures S9E–G**). However, it remained unclear how ICI therapies increased B_IR_-cell presence. The *in vitro* experiments showed that anti-PD-1 but not anti-CTLA4 antibody treatment could increase B_IR_ cells (**Figures 7H, I**). In addition, T cells pretreated with these antibodies failed to increase B_IR_ cells (**Figures 7H, I**). Therefore, anti-PD-1 antibodies directly induce B_IR_-cell formation, but not through an indirect effect of T cells.

**Figure 8:**
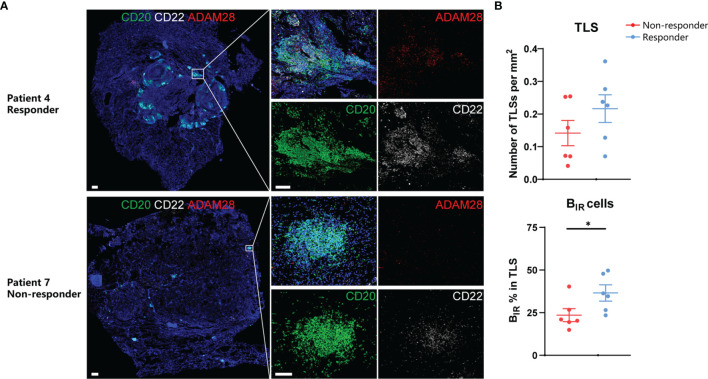
Prognosis value of B_IR_ cells in ICI-treated patients. **(A)** Representative immunofluorescence staining of B_IR_ cells for CD20 (green),CD22 (white), and ADAM28 (red) from NSCLC patients response or no response to ICI therapy.All DAPI staining is shown in blue. Scale bar = 100m. **(B)** Quantification of TLSs density (up) and B_IR_ cells percent in TLS (down) by immunohistochemistry and association with response to adjuvant ICI(n = 6 non-responders and 6 responders).Data are mean values ±SEM, and individual data points are shown.*P < 0.05.

On the basis of the results from transcriptome and animal experiments, we next assessed tumor samples histologically to gain insight into the density and distribution of B_IR_ cells as well as their relationship to TLSs in ICI-treated patients. We collected FFPE samples from 12 NSCLC patients treated with adjuvant checkpoint blockade (6 responders and 6 non-responders) (**Table S1**). The B_IR_-cell percentage in TLSs but not TLS density was significantly higher in responders than in non-responders (**Figure 8**), suggesting that B_IR_-cell infiltration in TLSs is predictive of the response to ICI when assessing pre-treatment samples. Findings between transcriptome and immunohistochemistry analysis were complementary, suggesting that B_IR_ cells indicate a favorable prognosis for patients with ICI therapy.

## Discussion

The role of B cells in tumor immunosurveillance has been studied to a much lesser extent than that of T cells. However, several studies indicate that B cells’ presence and functionality can be considered a significant prognostic factor in cancer, especially for patients treated with immunotherapy (50). A special B-cell subpopulation plays a major role in the immune microenvironment of ICI-treated patients, which we thus call B_IR_. In contrast to T cells often scattering within tumors, most B cells localize together to form tumor-associated immune aggregates of various complexity, ranging from small unorganized clusters to structured TLSs. The proportion of TLSs varies according to tumor types (48, 51). Consistently, in this paper, we also identified TLSs in breast cancer, RCC, skin SCC, and CRC. The presence of TLS has a favorable association with outcomes in almost all these cancer types, regardless of whether patients are treatment-naïve, or receive chemotherapy or immunotherapy (4).

The underlying mechanisms of how B cells in the TLSs promoted the response to ICI therapy require further investigation. It is speculated that B cells present tumor-associated antigens to T cells and thereby shape antigen-specific immune responses (11). In addition, B cells in TLSs undergo antigen-driven clonal amplification, somatic hypermutation, and affinity maturation *in situ* (52), and then differentiate into plasma cells, which secrete IgG and IgA to recognize multiple tumor antigens (10). CD40L expressed by T cells in TLSs supports the differentiation of mature B cells into memory B cells (53). As a subset of memory B cells, B_IR_ cells could potentially be involved in maintaining a long-term response against cancer.

In our study, ADAM28, CD180, and CD22 are identified as sensitive markers for B_IR_ cells in TLSs. ADAM28 is specifically expressed in B cells in human blood and may play a role in the adhesive and proteolytic events that occur during lymphocyte emigration. ADAM28 could also promote the differentiation of immature B cells to marginal zone B (MZB) cells in the spleen through the Notch2-RBP-Jκ pathway (54, 55). In addition, ADAM28 induces the shedding of soluble CD200 in B cells, which delivers immunomodulatory signals to suppress T cell-mediated anti-tumor responses (56). CD180, also known as RP105, is a TLR-like protein that physically associates with MD-1. The CD180/MD-1 complex is expressed on B lymphocytes, macrophages, and DCs. CD180 ligation on B cells could lead to its proliferation, resistance to apoptosis, and expression of CD86 through the Lyn-PI3K-BTK pathway, followed by the activation of protein kinase C b (PKCb), MAPKs (ERK, p38), and NFκB (57). Furthermore, ligation of CD180 significantly inhibits the response of B cells to type I interferon (58). In general, both ADAM28 and CD180 promote B-cell proliferation and differentiation. In contrast, CD22 (Siglec 2) is a regulatory receptor predominantly restricted to B cells, which inhibits both BCR and TLR signaling through four ITIMs (41). We found CD22 expressed in almost 73% of TLS B cells, which raised the question of what role CD22 plays in TLSs. It was reported that the antigen-driven differentiation of the naive B-cell repertoire in lymphoid tissues was divided into two consecutive phases. In phase 1, antigenic stimulation through BCR signaling induces naive B cells to differentiate into short-lived plasma cells in the B-cell follicles. In phase 2, B cells successfully receive CD40-mediated help from Tfh, survive, and exit the GC as long-lived plasma cells or memory B cells (59). We speculated that CD22 dampened BCR signaling to accelerate the transfer of B_IR_ cells from phase 1 to phase 2. Therefore, CD22 could promote B_IR_-cell differentiation into long-lived rather than short-lived plasma cells, enhancing long-term humoral immunity.

Cell–cell communication plays an important role in B cell-mediated immunity. Immature B cells receive CD40-mediated stimulation from T Follicular Helper (T_FH_) cells, generate germinal center (GC) B-cell responses, and exit the GC as long-lived plasma cells or memory B cells. Indeed, CD4^+^PD-1^hi^ICOS^+^CXCR5^+^ T_FH_ cells are present in close vicinity to GC B cells in TLSs (6, 60). They produce CXCL13 to facilitate the activation, proliferation, and differentiation of TLS B cells (60). An additional subset of PD-1^hi^ CD8^+^ T cells in the light B-cell zone also secrete CXCL13 in late-stage NSCLC tumors (61). These PD-1^hi^ T cells activate adaptive humoral responses in TLSs, explaining why ICI treatment may produce anti-tumor immunity in TLS-rich tumors (62). In addition, scattered DC-LAMP^+^ mature DCs infiltrate in the GC of TLSs, in close contact with B-cell follicles, allowing local antigen presentation to B cells (63, 64).

Literature proposed different gene signatures of TLSs, such as the 12-chemokine signature, which is related to the neogenesis of TLSs (4). Notably, variable degrees of expression levels of the 12 chemokines signature were observed in 30 cancer types of the TCGA cohorts, suggesting the pan-cancer distribution of TLSs (4). Furthermore, markers of several major cell populations in TLSs, such as T_FH_ cells, Th1 cells, B cells, and plasma cells, also constitute gene signatures of TLSs (4). Therefore, in our study, we identified novel B-cell markers for the detection of TLSs. Gene signatures of both B_IR_ cells and TLSs predict the favorable prognosis of ICI-treated patients.

As a limitation, it should be mentioned that RNA-seq projects and validation datasets in this study are retrospective. In addition, the concrete mechanism of how B_IR_ cells promoted tumor response to ICI therapy was incompletely understood. We found that ICI therapy increased B_IR_ cells with a memory phenotype and that B_IR_ cells were increased in TLS of ICI-responsive patients. However, it remains unclear whether B_IR_ cells contribute to an effective T-cell response after ICI therapy and how the TLS environment affects B_IR_-cell function.

In the future, prospective studies could be designed to validate the correlation between B_IR_ cells and improved prognosis under ICI therapy. Further studies are required to explore how B_IR_ cells independently contribute to anti-tumor immune function in the context of ICI therapy.

## Conclusion

In summary, our paper reported the presence of a novel CD20^+^CD22^+^ADAM28^+^ B-cell subpopulation within TLS in ICI responders, which we called ICI‐Responsive B cells (B_IR_). These B cells adopt a specialized memory phenotype with increased activation, potentially contributing to the anti‐tumor responses. Indeed, the presence of B_IR_ cells in tumors correlated with response to PD-1 therapy, and their depletion in melanoma-bearing mice inhibited the response to PD-1. Thus, B_IR_ cells could be used as a biomarker to predict which patients are more likely to benefit from ICI. These findings provide insights into new therapeutic approaches to enhance responses to ICI.

## Data Availability Statement

The raw data supporting the conclusions of this article will be made available by the authors, without undue reservation.

## Ethics Statement

The studies involving human participants were reviewed and approved by the Ethical Committee of the Tongji Medical College of Huazhong University of Science and Technology. The patients/participants provided their written informed consent to participate in this study. The animal study was reviewed and approved by Institutional Animal Care and Use Committee-approved protocols at Tongji Medical College of Huazhong University of Science and Technology.

## Author Contributions

ZW, JJZ, and YX contributed equally. Conception and design: ZW and TH. Acquisition of data (provided required samples and clinical information): JM, JZ, and FD. Animal experiments: ZW, YX, and ZX. Cellular experiments: XQZ and YX. Analysis and interpretation of data (statistical analysis and bioinformatics analysis): ZW and JJZ. Writing and/or revision of the manuscript: ZW, XWZ, and PL. Study supervision: XWZ, PL, and TH. All authors contributed to the article and approved the submitted version.

## Conflict of Interest

The authors declare that the research was conducted in the absence of any commercial or financial relationships that could be construed as a potential conflict of interest.

## Publisher’s Note

All claims expressed in this article are solely those of the authors and do not necessarily represent those of their affiliated organizations, or those of the publisher, the editors and the reviewers. Any product that may be evaluated in this article, or claim that may be made by its manufacturer, is not guaranteed or endorsed by the publisher.
