# Supplementary material for: CD20+CD22+ADAM28+ B Cells in Tertiary Lymphoid Structures Promote Immunotherapy Response
Source: Front Immunol. 2022 May 11;13:865596. doi: 10.3389/fimmu.2022.865596 (PMC9130862; doi:10.3389/fimmu.2022.865596)
Supplement: Supplementary file 11 [file Table_1.pdf]

Sample ID	Diagnosis age	Sex	Pathologic tumor	Lymph node involvement	Sample type	Cancer type	Histology	Evaluation before ICI therapy	ICI type	ICI drug	Other drugs	iRECIST	Best overall response
WHUH-01	55	Male	IIIb	Yes	Primary	NSCLC	Invasive adenocarcinoma	Brain metastasis, Bone metastasis	anti-PD-1	Camrelizumab	No	iSD	NR
WHUH-02	50	Female	IIIa	Yes	Primary	NSCLC	Invasive adenocarcinoma	Lung recurrence	anti-PD-1	Camrelizumab	Apatinib, Pemetrexed,	iCPD	NR
WHUH-03	66	Male	IIb	Yes	Primary	NSCLC	Invasive adenocarcinoma	Liver metastasis	anti-PD-1	Sintilimab	Bevacizumab, Taxanes, Platinum	iCR	R
WHUH-04	67	Male	IIIa	Yes	Primary	NSCLC	Invasive adenocarcinoma	Lymph node metastasis	anti-PD-1	Camrelizumab	No	iPR	R
WHUH-05	64	Male	IIb	Yes	Primary	NSCLC	Invasive adenocarcinoma	Brain metastasis	anti-PD-1	Camrelizumab	Temozolomide	iUPD	NR
WHUH-06	68	Male	Ib	No	Primary	NSCLC	Invasive adenocarcinoma	Lung recurrence, Lymph node	anti-PD-1	Sintilimab	Taxanes, Platinum	iPR	R
WHUH-07	49	Male	IIIa	Yes	Primary	NSCLC	Invasive adenocarcinoma	Lung recurrence	anti-PD-1	Camrelizumab	No	iUPD	NR
WHUH-08	43	Male	IIIa	Yes	Primary	NSCLC	Invasive adenocarcinoma	Lung recurrence	anti-PD-1	Sintilimab	Bevacizumab, Pemetrexed,	iCR	R
WHUH-09	67	Male	IIIa	Yes	Primary	NSCLC	Squamous cell carcinoma	Bone metastasis	anti-PD-1	Pembrolizumab	Taxanes, Platinum	iSD	NR
WHUH-10	52	Male	Ia3	No	Primary	NSCLC	Invasive adenocarcinoma	Lung recurrence	anti-PD-1	Pembrolizumab	Bevacizumab, Taxanes, Platinum	iPR	R
WHUH-11	72	Male	Ib	No	Primary	NSCLC	Squamous cell carcinoma	Lung recurrence	anti-PD-1	Pembrolizumab	No	iUPD	NR
WHUH-12	66	Male	IIIa	Yes	Primary	NSCLC	Squamous cell carcinoma	Lymph node metastasis	anti-PD-1	Pembrolizumab	Taxanes, Platinum	iPR	R
