# Supplementary material for: CD20+CD22+ADAM28+ B Cells in Tertiary Lymphoid Structures Promote Immunotherapy Response
Source: Front Immunol. 2022 May 11;13:865596. doi: 10.3389/fimmu.2022.865596 (PMC9130862; doi:10.3389/fimmu.2022.865596)
Supplement: Supplementary file 12 [file Table_2.pdf]

Supplementary Table 2. PRECOG z-scores of each gene in different kinds of cancers histology, related to Fig.5D

**Gene**	**CD20**	**CD22**	**ADAM28**	**EBF1**	**GNG7**	**ATP2A3**	**MEF2C**	**CPNE5**
Unweighted_meta-Z_of_all_cancers	-6.4044	-2.8518	-3.6253	-5.2667	-3.7699	-4.0285	-5.3787	-4.0618
Adrenocortical_cancer	0.859	-0.154	-1.498	-3.403	-1.137	1.163	-1.467	-0.159
Bladder_cancer	-0.41	-0.051	-1.752	-0.349	-1.378	-2.229	-2.093	1.117
Brain_cancer_Astrocytoma	-0.757	1.158	-0.235	0.264	0.048	1.411	-1.336	-3.121
Brain_cancer_Glioblastoma	0.958	-0.098	0.171	-2.359	0.132	-1.005	0.46	-0.961
Brain_cancer_Glioma	-2.27	-1.002	2.31	-3.568	0.117	-0.144	0.34	-0.692
Brain_cancer_Medulloblastoma	0.138	0.067	0	0	0	-1.13	-0.722	0
Brain_cancer_Meningioma	0.333	-1.162	0.427	-2.166	1.767	-1.067	-0.14	0.452
Brain_cancer_Neuroblastoma	-0.783	-1.602	-3.366	-11.34	0.009	-4.904	-3.987	-5.602
Breast_cancer	-1.302	-2.538	-3.012	-3.093	-2.065	2.052	-1.637	0.449
Colon_cancer	-1.304	0.217	1.402	1.838	0.053	-0.739	0.853	-0.217
Gastric_cancer	-0.684	-0.042	-0.718	0.217	-2.06	-0.774	-1.722	-0.579
Germ_cell_tumors	-3.907	-3.047	-0.889	-2.154	-1.908	-3.366	-2.329	-4.131
Head_and_neck_cancer	-2.775	-2.209	-2.196	0.645	-1.769	-0.356	-0.728	-0.142
Head_and_neck_cancer_Hypopharyngeal_cancer	-1.578	0.169	1.292	0	1.449	0.092	-1.029	0
Head_and_neck_cancer_Oesophageal_cancer	1.213	0	0	0.17	0	1.524	0.668	0
Head_and_neck_cancer_Oral_SCC	-0.933	-1.103	-0.329	-0.67	0.532	-0.64	-0.832	-0.064
Hematopoietic_cancer_AML	-2.75	-0.008	2.971	0.516	-1.472	-1.062	4.929	-1.322
Hematopoietic_cancer_B_ALL	-0.301	-1.1	0.178	-0.854	0.294	-0.992	1.874	-0.628
Hematopoietic_cancer_Burkitt_lymphoma	0.578	-1.933	0.242	1.702	0.993	-0.858	-1.331	0
Hematopoietic_cancer_CLL	0.619	1.163	-1.814	-0.389	0.007	0.934	-1.219	1.978
Hematopoietic_cancer_DLBCL	-3.94	-2.784	-1.918	-3.608	-0.48	-0.189	-7.66	-3.14
Hematopoietic_cancer_FL	-1.476	-0.389	-0.787	-2.551	-2.373	-1.829	-2.424	1.057
Hematopoietic_cancer_Mantle_cell_lymphoma	-2.003	3.203	-1.087	-0.299	0	-1.297	0.907	0.478
Hematopoietic_cancer_Multiple_myeloma	-0.313	-0.542	-1.848	-1.13	-3.863	0.842	0.469	-1.869
Kidney_cancer	0	-0.687	0	0	0	0	-0.224	0
Liver_cancer	-0.673	0.404	1.068	0.605	-0.787	-0.457	-0.779	-1.561
Liver_cancer_Primary	0.769	0	0	1.46	2.05	-0.422	-1.386	0
Lung_cancer_ADENO	-4.584	-5.227	0.384	-2.507	-5.813	-0.322	-4.392	-0.307
Lung_cancer_LCC	0.204	0.126	0.846	-1.006	-0.819	0.946	-0.334	0.386
Lung_cancer_SCC	-2.582	0.513	-1.833	-0.389	0.617	-2.24	-1.055	-0.72
Lung_cancer_SCLC	-0.944	0.445	-1.047	0.644	-1.406	-0.293	-0.572	-0.109
Melanoma	-1.388	-1.363	-1.72	-1.352	-2.223	-1.912	-1.065	-1.47
Melanoma_Metastasis	-3.176	-2.262	-3.048	-2.789	0.972	-3.269	-1.839	-1.499
Mesothelioma	-2.673	1.011	-0.307	0	0.027	-0.504	-0.142	0
Ovarian_cancer	-0.812	1.736	-2.937	1.631	0.135	-1.417	-0.599	-2.106
Pancreatic_cancer	0.496	1.017	0.242	-0.322	0.244	-0.59	-0.002	-0.738
Prostate_cancer	-0.317	-1.539	0.685	-0.002	-0.996	0.855	-0.354	0.508
Sarcoma_Ewing_sarcoma	-0.917	1.67	-1.373	1.138	-0.749	-1.002	-1.477	-1.131
Sarcoma_Osteosarcoma	-0.609	0.132	-1.144	2.578	-1.691	0.034	0.786	0.477
